# Why healthy life expectancy estimates diverge: insights from survey-based, administrative, and model-based metrics across Japanese prefectures, 2001–2019: a comparative ecological study

**DOI:** 10.1186/s12963-026-00488-z

**Published:** 2026-06-10

**Authors:** Shuhei Nomura, Akifumi Eguchi, Daisuke Yoneoka, Hana Tomoi, Lisa Yamasaki, Yuta Tanoue, Takayuki Kawashima, Aoi Kataoka, Yuri Ito, Naoki Kondo

**Affiliations:** 1 Global Health Policy Lab, International Research Institute of Disaster Science (IRIDeS), Tohoku University, 468-1 Aoba, Aramaki, Aoba-ku, Sendai, Miyagi, 980-8572 Japan; 2 Department of Health Policy and Management, School of Medicine, Keio University, Tokyo, Japan; 3 Center for Preventive Medical Sciences, Chiba University, Chiba, Japan; 4 Infectious Disease Surveillance Center, National Institute of Infectious Diseases, Japan Institute for Health Security, Tokyo, Japan; 5 Department of Global Health Policy, Graduate School of Medicine, The University of Tokyo, Tokyo, Japan; 6 London School of Hygiene and Tropical Medicine, Department of Infectious Disease Epidemiology, Faculty of Epidemiology and Population Health, London, UK; 7 The School of Tropical Medicine and Global Health, Nagasaki University, Nagasaki, Japan; 8 Department of Environmental Health, Harvard T.H. Chan School of Public Health, Boston, MA USA; 9 Department of Emergency Medicine and Critical Care, Japan Institute for Health Security, Tokyo, Japan; 10 Faculty of Marine Technology, Tokyo University of Marine Science and Technology, Tokyo, Japan; 11 School of Computing, Institute of Science Tokyo, Tokyo, Japan; 12 Department of Medical Statistics, Osaka Medical and Pharmaceutical University, Research & Development Center, Osaka, Japan; 13 Department of Molecular Epidemiology, Future Medical Science, Kobe University Graduate School of Medicine, Hyogo, Japan; 14 Department of Social Epidemiology, Graduate School of Medicine and School of Public Health, Kyoto University, Kyoto, Japan

**Keywords:** Healthy life expectancy, Global burden of disease, Japan, Subnational analysis, Health metrics

## Abstract

**Background:**

Healthy life expectancy (HALE) can be measured using different approaches, yet estimates may diverge. Understanding divergence sources could help identify which health dimensions current systems capture or miss. However, systematic subnational comparisons remain scarce.

**Methods:**

We conducted a comparative ecological study across Japan’s 47 prefectures from 2001 to 2019. Three Ministry of Health, Labour and Welfare (MHLW) metrics—disability-free life expectancy based on activity limitation (DFLE-AL) and life expectancy in subjective health (LE-SH) derived from national surveys, and disability-free life expectancy based on activities of daily living (DFLE-ADL) derived from long-term care insurance records—were obtained from MHLW. Model-based HALE from the Global Burden of Disease (GBD) 2023 Study was extracted for corresponding years. We assessed cross-sectional concordance and examined associations with disease burden, risk factors, and socioeconomic indicators using fixed-effects panel regression.

**Results:**

Over 2001–2019, DFLE-AL and LE-SH correlated with GBD HALE among males (r = 0.33 to 0.65) but weakly among females (r =  − 0.11 to 0.32); DFLE-ADL showed concordance for both sexes (r = 0.66 to 0.96). GBD HALE exceeded MHLW estimates in females (0.53 years for DFLE-AL, 0.23 for LE-SH) but not males (− 0.18, − 0.14). In fixed-effects analysis of MHLW-minus-GBD differences, mental disorders showed negative associations with DFLE-AL and LE-SH differences among males; high BMI and musculoskeletal disorders showed positive associations with DFLE-AL differences. Among females, population ageing showed positive associations with DFLE-AL, LE-SH, and DFLE-ADL differences.

**Conclusions:**

MHLW metrics and GBD HALE capture complementary health dimensions and should not be treated as interchangeable. The consistent sex difference in concordance indicates that divergence between HALE metrics reflects differences in which health dimensions are emphasised—self-reported health states, objectively assessed functional status, or disease-based disability modelling—rather than a single, uniform construct of population health.

**Supplementary Information:**

The online version contains supplementary material available at 10.1186/s12963-026-00488-z.

## Introduction

Healthy life expectancy (HALE) has become a cornerstone metric in global health policy. The Sustainable Development Goals (SDGs) framework monitors HALE as an indicator of population well-being [1]. Across high- and low-income settings, governments have adopted targets to extend healthy lifespan [2]. For example, the UK aims to add 5 healthy years by 2035, Saudi Arabia plans to increase HALE by 6 years by 2030, and China to raise HALE to 79 years by 2030 [3]. These ambitions reflect a broad policy consensus that simply living longer is not enough – health systems must strive to ensure those added years are lived in good health [2]. Yet a fundamental measurement challenge underlies this goal: multiple definitions of HALE coexist, each derived from different data and methods. These approaches are grounded in distinct conceptualisations of health—self-perceived lived experience, administratively certified functional independence, and model-based epidemiological disability—which may not align even when applied to the same population. Whether these metrics identify the same populations as “healthy” or “unhealthy” remains an unanswered question with global relevance. Understanding why estimates diverge could reveal which health dimensions each approach captures or misses—knowledge essential for developing more effective strategies to extend healthy life.

Underlying this policy attention is a long-standing debate about how additional years of life relate to years lived in good health. The compression-of-morbidity hypothesis posits that, as life expectancy lengthens, disease and disability become increasingly concentrated into a shorter span at the end of life [4]. The expansion-of-morbidity view holds the opposite, with longer survival accompanied by more years lived with functional limitation; intermediate accounts envisage a dynamic equilibrium in which severe disability compresses even as milder limitations expand [5]. HALE is the principal empirical indicator used to adjudicate between these scenarios, but whether different HALE definitions yield consistent answers depends critically on how “health” is operationalised across measurement approaches [6].

HALE estimation approaches vary widely in their data sources and methods. Survey-based approaches, used by many national agencies, directly ask individuals about functional limitations or self-rated health, capturing what populations report about their lived health experiences [7]. For instance, the EU tracks “healthy life years” (years without moderate or severe disability) and the share of people in good or very good self-perceived health as parallel indicators [7]. Such metrics are often derived using the Sullivan method, combining life tables with prevalence of disability or suboptimal self-rated health from sample surveys [8]. Model-based approaches, exemplified by the Global Burden of Disease (GBD) Study’s HALE, synthesize epidemiological data on hundreds of diseases and injuries through statistical modeling [8, 9]. GBD HALE is computed by subtracting years lived with disability (YLDs), quantified using internationally derived disability weights covering hundreds of diseases and conditions, from life expectancy within a standardised framework [10–12]. GBD’s standardized methods facilitate comparisons across countries and over time [13]. Several countries now have multiple national HALE metrics—including survey-based, administrative data-based, and model-based estimates—available for subnational comparison [13, 14]. Whether these different approaches yield concordant assessments of regional health – or diverge in ways that could mislead policy – is poorly understood.

The choice of HALE metric is not merely technical; it shapes which regions are identified as health priorities and how resources are allocated. When metrics derived from different data sources and methodologies rank areas differently, policies anchored to a single approach may fail to capture populations deemed unhealthy under alternative frameworks, thereby embedding measurement bias into resource allocation. This risk is especially pronounced in subnational planning, where systematic differences in demographic structure, disease profiles, and health reporting practices provide a structural basis for metric divergence rather than random variation. Despite the proliferation of HALE metrics, few studies have systematically compared nationally defined survey-based metrics with global model-based estimates at the subnational level [15, 16]. Importantly, existing work has largely focused on absolute differences in levels, rather than on concordance in regional ranking or the structural factors associated with divergence between metrics. One local analysis in Spain found that multistate morbidity-based HALE was 8–9 years lower than survey-based HALE, highlighting how method differences can yield large outcome gaps [16]. The factors that may drive divergence between these fundamentally different measurement approaches – including sex, disease burden, risk factors, and socioeconomic conditions – remain poorly understood.

Japan offers an ideal case study to address this knowledge gap. As a demographic frontrunner, Japan’s experience in reconciling diverse health metrics serves as a vital blueprint for other rapidly ageing nations navigating the transition from a focus on extending life to ensuring that those additional years are lived in good health. As the world’s most aged country with universal health coverage, Japan tracks multiple nationally defined HALE metrics for policy purposes alongside GBD estimates for global comparison. Its 47 prefectures vary widely in demographic and socioeconomic profiles, mirroring the regional diversity within many countries. Crucially, Japan’s Ministry of Health, Labour and Welfare (MHLW) has adopted HALE as a primary health target. Health Japan 21, a ten-year national health promotion initiative that sets quantitative targets across multiple health domains, explicitly aims to extend HALE as well as total life expectancy [17, 18]. At the same time, GBD estimates are widely used for international comparison of Japan’s health status, in line with the SDG framework for monitoring population health [9, 17, 19]. This juxtaposition of nationally defined and model-based metrics in one country provides a unique opportunity to examine their concordance. Our study thus compares three MHLW HALE metrics – with GBD 2023 HALE across Japan’s prefectures from 2001 to 2019. Rather than asking whether these metrics differ, we examine under what conditions divergence emerges, and what structural and epidemiological conditions are associated with it. We evaluate whether concordance between approaches varies by sex, and investigate disease burden, risk factor, and socioeconomic correlates of metric divergence. Specifically, we (1) assess cross-sectional concordance across prefectures, (2) examine correlates of divergence at a given time point, and (3) analyse within-prefecture changes over time using fixed-effects models. Our goal is not to declare a “correct” metric – all capture important health dimensions – but to identify conditions under which nationally defined and model-based approaches converge or diverge. Understanding these conditions is essential because persistent divergence between global monitoring tools and national policy instruments may undermine local trust in international health targets; identifying what drives divergence can help policymakers interpret discordant signals and allocate resources more equitably. This evidence can inform the appropriate use and interpretation of HALE indicators in health policy.

## Methods

### Study design and setting

We conducted a comparative ecological study of HALE metrics across Japan’s 47 prefectures. Three domestically used HALE definitions developed by MHLW – disability-free life expectancy based on activity limitation (DFLE-AL; years lived without activity limitation), life expectancy in subjective health (LE-SH; years lived in self-perceived good health), and disability-free life expectancy based on activities of daily living (DFLE-ADL; years lived with independence in activities of daily living) [20] – were compared against HALE from GBD 2023. All analyses were stratified by sex.

### Data sources

#### Healthy life expectancy metrics

Prefecture-level DFLE-AL, LE-SH, and DFLE-ADL were obtained from MHLW [21]. DFLE-AL and LE-SH were available for seven time points (2001, 2004, 2007, 2010, 2013, 2016, and 2019); DFLE-ADL was available for four time points (2010, 2013, 2016, and 2019). DFLE-AL and LE-SH are derived from the Comprehensive Survey of Living Conditions and are estimated at three-year intervals when the large-scale survey including health questionnaires is conducted: DFLE-AL measures years lived without limitations in activities of daily living based on disability status, capturing any self-reported restriction in daily activities, while LE-SH measures years lived in self-rated good health based on subjective health responses [20]. DFLE-ADL, in contrast, is derived from administrative data from Japan’s Long-Term Care Insurance (LTCI) system, a mandatory social insurance programme established in 2000 that certifies care-need levels for adults aged 65 years and older (or adults aged 40–64 with specified diseases); DFLE-ADL measures years lived without care-need certification of level 2 or higher, where level 2 indicates requirement for assistance with basic activities of daily living such as bathing, dressing, or eating [20]. This metric reflects institutional recognition of dependency rather than the presence of functional limitation per se, and therefore represents a higher threshold for defining disability [14]. All three MHLW metrics are calculated using Sullivan’s method, integrating life tables with age-specific prevalence of activity limitation, poor self-rated health, or care-need certification, respectively [22, 23]. Prefecture-level HALE was extracted from GBD 2023 for corresponding years [24]. GBD HALE is computed by subtracting YLDs from life expectancy, with YLDs derived from disease-specific prevalence estimates and standardised disability weights; detailed methodology has been described elsewhere [24]. In contrast to the MHLW metrics that directly capture self-reported health states or objectively assessed functional status, GBD HALE is a composite indicator constructed from epidemiological models covering hundreds of causes.

#### Covariates

To examine factors associated with divergence between MHLW and GBD HALE metrics, we assembled covariates from three domains: (1) disease burden indicators (age-standardised DALYs for neoplasms, cardiovascular diseases, musculoskeletal disorders, neurological disorders, and mental disorders from GBD 2023); (2) risk factor exposures (summary exposure values for tobacco use, high fasting plasma glucose, high systolic blood pressure, high BMI, and dietary risks from GBD 2023); and (3) socioeconomic and healthcare indicators (per capita medical expenditure, number of nursing-home facilities per 100,000 population aged ≥ 65 years, labour force participation rate, university graduation rate, annual income, and proportion aged ≥ 75 years from Japanese government sources). Detailed variable definitions, rationale for selection, data sources, and data availability are provided in the Supplementary Text.

### Statistical analysis

We computed the difference between each MHLW metric and GBD HALE (e.g., DFLE-AL minus GBD HALE) to quantify divergence between measurement approaches. This operationalisation treats divergence as a directional discrepancy without implying that either metric constitutes a gold standard. This difference served as the primary outcome variable for subsequent analyses.

In cross-sectional analyses using 2019 data, we examined correlations between MHLW and GBD estimates through scatter plots with fitted regression lines and 95% confidence intervals. Pearson correlation coefficients with two-sided p-values were calculated separately for males and females. We further assessed correlations between the MHLW-GBD difference and each set of covariates (disease burden, risk factors, and socioeconomic indicators) using the same approach.

For longitudinal analysis, we employed fixed-effects panel regression models with time-interaction terms across seven time points (2001, 2004, 2007, 2010, 2013, 2016, and 2019) for DFLE-AL and LE-SH, and across four time points (2010, 2013, 2016, and 2019) for DFLE-ADL. Models included prefecture and year fixed effects to control for time-invariant prefectural characteristics and secular trends. To capture the time-variant effects of covariates, we included interaction terms between each covariate and year indicators. Cluster standard errors at the prefecture level were calculated to account for within-prefecture correlation. We estimated two separate model sets reflecting conceptually distinct domains and different policy implications: the first included disease burden indicators and risk factor exposures, representing epidemiological factors amenable to health promotion and disease prevention interventions; the second included socioeconomic and healthcare indicators, representing structural and contextual factors addressable through health system and social policies. This separation also served to avoid overfitting and multicollinearity given large number of parameters. Separate models were estimated for DFLE-AL, LE-SH, and DFLE-ADL outcomes, and for males and females. Results are presented as time-varying coefficient plots with 95% confidence intervals. All analyses were conducted using Stata version 17 (StataCorp, College Station, TX, USA). Statistical significance was defined as p < 0.05 (two-sided).

### Large language model use

Claude (Anthropic) was used to assist with language editing, code review, and manuscript refinement. All outputs were critically reviewed and verified by the authors, who take full responsibility for the content. Use of this tool is documented here in accordance with journal policy on large language models.

## Results

Table 1 presents descriptive statistics for HALE metrics and covariates in 2001, 2010, and 2019. All four HALE metrics increased substantially over the study period for both sexes. For context, GBD life expectancy at birth in 2019 averaged 81.4 years for males and 87.6 years for females across prefectures, indicating a gap of approximately 9 and 12 years, respectively, between total survival and GBD HALE. Age-standardised disease burden (DALYs per 100,000) from neoplasms and cardiovascular diseases declined markedly over this period, while mental disorder burden increased for both sexes. Among age-standardised risk factor exposures, tobacco and high blood pressure declined, while high BMI increased. Per capita medical expenditure and number of nursing-home facilities increased substantially, and the proportion of population aged ≥ 75 years approximately doubled over the study period.

**Table 1:** Healthy life expectancy metrics and covariates across 47 Japanese prefectures, 2001, 2010, and 2019

	**Males**			**Females**		
	**2001**	**2010**	**2019**	**2001**	**2010**	**2019**
***Healthy life expectancy, years***						
Life expectancy (GBD)	78.0 (0.6)	79.6 (0.6)	81.4 (0.6)	85.1 (0.4)	86.5 (0.4)	87.6 (0.4)
GBD HALE	69.1 (0.5)	70.4 (0.5)	72.2 (0.6)	73.6 (0.4)	74.4 (0.3)	75.5 (0.4)
DFLE-AL	69.2 (0.9)	70.4 (0.7)	72.6 (0.6)	72.9 (0.7)	73.7 (0.8)	75.6 (0.7)
LE-SH	69.4 (0.9)	69.9 (0.8)	73.1 (0.6)	73.1 (0.7)	73.4 (0.8)	76.6 (0.6)
DFLE-ADL	NA	78.1 (0.6)	79.8 (0.7)	NA	83.2 (0.4)	84.1 (0.5)
Disease burden, DALYs per 100 000*						
Neoplasms	4531.3 (285.8)	3940.7 (254.5)	3394.2 (241.3)	2454.3 (118.4)	2229.5 (112.5)	2097.2 (144.4)
Cardiovascular diseases	3339.7 (258.4)	2797.4 (266.9)	2399.1 (237.5)	1714.7 (135.2)	1332.4 (114.4)	1118.1 (104.5)
Musculoskeletal disorders	2027.1 (18.7)	2096.4 (17.6)	2087.1 (16.4)	3017.6 (60.6)	3119.5 (61.1)	3034.4 (51.0)
Neurological disorders	839.9 (20.9)	882.5 (21.4)	969.7 (27.6)	1272.9 (34.1)	1288.4 (32.7)	1348.5 (35.0)
Mental disorders	1584.7 (34.2)	1692.6 (35.2)	1860.9 (38.7)	1609.7 (68.8)	1695.1 (73.5)	1898.8 (83.7)
Risk factor exposure, SEV*						
Tobacco	40.3 (1.9)	33.7 (2.0)	29.3 (1.9)	27.4 (1.8)	22.4 (1.8)	18.0 (1.6)
High fasting plasma glucose	16.1 (2.0)	15.8 (2.0)	15.9 (2.0)	14.7 (2.0)	13.8 (1.9)	13.5 (1.8)
High systolic blood pressure	35.9 (2.8)	30.5 (1.9)	28.9 (1.4)	24.5 (2.3)	17.9 (1.3)	17.8 (1.1)
High body-mass index	12.2 (1.1)	13.4 (1.2)	15.0 (1.2)	10.8 (0.6)	11.3 (0.6)	12.5 (0.6)
Dietary risks	40.8 (0.7)	40.1 (0.7)	41.2 (0.7)	37.1 (1.0)	35.2 (0.8)	36.3 (0.8)
***Socioeconomic indicators***						
Per capita medical expenditure, thousand yen	247.7 (29.8)	305.3 (34.8)	367.3 (39.0)	247.7 (29.8)	305.3 (34.8)	367.3 (39.0)
Number of nursing-home facilities‡	37.0 (7.6)	48.1 (12.1)	82.4 (30.6)	37.0 (7.6)	48.1 (12.1)	82.4 (30.6)
Labour force participation, %	74.0 (2.2)	69.7 (2.2)	64.8 (3.0)	48.8 (2.7)	47.8 (2.3)	49.3 (2.5)
University graduation rate, %	18.7 (5.2)	22.5 (6.0)	26.6 (6.5)	5.9 (2.2)	8.8 (3.2)	12.3 (4.3)
Annual income, thousand yen	5136.3 (569.0)	4723.3 (541.6)	5051.4 (562.7)	3272.6 (343.0)	3207.8 (309.5)	3563.2 (351.8)
Population aged ≥ 75 years, %	6.3 (1.2)	9.6 (1.6)	12.7 (1.3)	10.7 (1.9)	15.1 (2.6)	18.7 (2.4)

Data are mean (standard deviation) across 47 prefectures. Healthy life expectancy metrics are expressed in years. Prefectural life expectancy is shown only for GBD [24] because MHLW prefectural life tables are published at five-year intervals and 2001 and 2019 values are not directly available. *Disease burden indicators and risk factor exposures are age-standardised estimates from Global Burden of Disease Study 2023. DALYs = disability-adjusted life-years per 100 000 population. SEV = summary exposure value (scale 0–100). †Per capita medical expenditure includes public funding, insurance premiums, and out-of-pocket payments. ‡Per 100 000 population aged ≥ 65 years. HALE = healthy life expectancy. DFLE-AL = disability-free life expectancy based on activity limitation. LE-SH = life expectancy in subjective health. DFLE-ADL = disability-free life expectancy based on activities of daily living

### Correlation between MHLW and GBD healthy life expectancy estimates

On average across all prefectures over the study period, GBD HALE was slightly higher than DFLE-AL and LE-SH in females (mean difference over 2001–2019: 0.53 [SD 0.89] years for DFLE-AL and 0.23 [SD 1.15] years for LE-SH), whereas it was slightly lower in males (mean difference: − 0.18 [SD 0.66] years for DFLE-AL and − 0.14 [SD 0.83] years for LE-SH). DFLE-ADL was substantially higher than GBD HALE across all years (2010–2019), with mean differences of 7.63 (SD 0.25) years among males and 8.70 (SD 0.31) years among females, reflecting the less restrictive definition of disability used in this administrative data-based metric. DFLE-AL and LE-SH were strongly correlated with each other across all years for both sexes (males: r = 0.76–0.89, p < 0.001; females: r = 0.66–0.83, p < 0.001). DFLE-ADL showed weaker correlations with DFLE-AL (males: r = 0.44–0.55; females: r = 0.03–0.20) and LE-SH (males: r = 0.47–0.62; females: r = 0.23–0.46), indicating that these three MHLW metrics capture distinct dimensions of health.

**Fig. 1:**
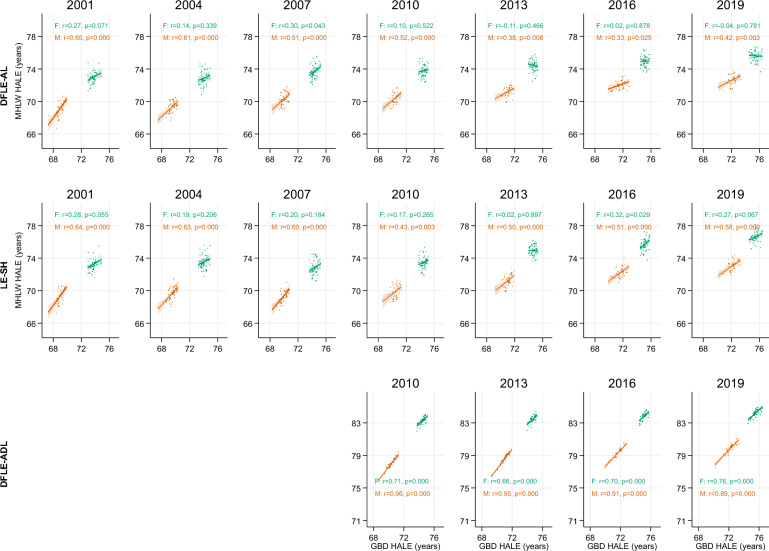
Correlation between MHLW and GBD 2023 healthy life expectancy (HALE) estimates across Japanese prefectures, 2001–2019. Scatter plots show the relationship between prefecture-level estimates from the Ministry of Health, Labour and Welfare (MHLW; y-axis) and the Global Burden of Disease Study 2023 (GBD 2023; x-axis) for disability-free life expectancy based on activity limitation (DFLE-AL; first row), life expectancy in subjective health (LE-SH; second row), and disability-free life expectancy based on activities of daily living (DFLE-ADL; third row; data available from 2010 onwards). Each point represents one prefecture. Lines indicate fitted regression with 95% confidence intervals (shaded areas). Pearson correlation coefficients (r) and p-values are shown separately for females (green) and males (orange)

Figure 1 presents scatter plots comparing prefecture-level MHLW estimates (DFLE-AL, LE-SH, and DFLE-ADL) with GBD HALE. A marked sex difference emerged in the concordance between measurement approaches. Among males, we observed consistent positive correlations between all three MHLW metrics and GBD HALE across all years. For DFLE-AL, correlations ranged from r = 0.33 (p = 0.025) in 2016 to r = 0.65 (p < 0.001) in 2001. For LE-SH, correlations were similarly moderate, ranging from r = 0.43 (p = 0.003) in 2010 to r = 0.64 (p < 0.001) in 2001. DFLE-ADL showed remarkably strong correlations with GBD HALE among males, ranging from r = 0.89 (p < 0.001) in 2019 to r = 0.96 (p < 0.001) in 2010. These findings indicate that prefectures ranking higher on GBD HALE also tended to rank higher on MHLW metrics among males. In contrast, among females, correlations between DFLE-AL and LE-SH and GBD HALE were substantially weaker and largely non-significant, with coefficients ranging from r =  − 0.11 (p = 0.466) to r = 0.32 (p = 0.029) for DFLE-AL and from r = 0.02 (p = 0.897) to r = 0.32 (p = 0.029) for LE-SH. DFLE-ADL, however, showed strong correlations with GBD HALE even among females, ranging from r = 0.66 (p < 0.001) in 2013 to r = 0.76 (p < 0.001) in 2019. This sex difference in concordance was consistent across all years for DFLE-AL and LE-SH, but not for DFLE-ADL, suggesting that the administrative data-based metric captures population health patterns more consistently across sexes than the survey-based metrics.

### Cross-sectional associations with disease burden, risk factors, and socioeconomic indicators

**Fig. 2:**
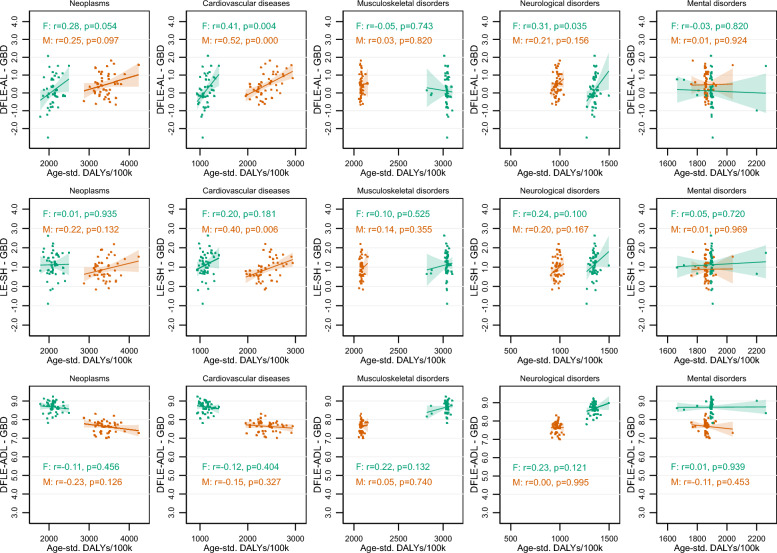
Cross-sectional correlations between MHLW-GBD 2023 differences and disease burden indicators, 2019. Scatter plots show the relationship between the difference in HALE estimates (MHLW minus GBD 2023; y-axis) and age-standardised disability-adjusted life-years (DALYs) per 100,000 population from GBD 2023 (x-axis) for five leading disease categories in Japan. Top row: DFLE-AL minus GBD 2023 HALE; bottom row: LE-SH minus GBD 2023 HALE. Positive values indicate MHLW estimates exceed GBD 2023 estimates. Lines indicate fitted regression with 95% confidence intervals. Pearson correlation coefficients and p-values are shown for males (orange) and females (green)

Figure 2 shows cross-sectional correlations between the MHLW-GBD difference and disease burden indicators using 2019 data. Cardiovascular disease DALYs showed the strongest associations with DFLE-AL and LE-SH differences: prefectures with higher cardiovascular disease burden had larger positive differences between these metrics and GBD HALE (DFLE-AL: males r = 0.52, p < 0.001; females r = 0.41, p = 0.004; LE-SH: males r = 0.40, p = 0.006; females r = 0.20, p = 0.181). This pattern indicates that in prefectures with high cardiovascular burden, MHLW estimates exceeded GBD estimates by a greater margin. Neoplasm DALYs showed moderate positive correlations with DFLE-AL among both sexes (males r = 0.25, p = 0.097; females r = 0.28, p = 0.054) and with LE-SH among males (r = 0.22, p = 0.132). Neurological disorder DALYs showed a significant positive correlation with DFLE-AL among females (r = 0.31, p = 0.035). In contrast, musculoskeletal disorders and mental disorders showed no significant associations with either of these metrics for either sex, despite being leading contributors to disability burden in Japan. For DFLE-ADL, no disease burden indicator showed significant associations with the MHLW-GBD difference, likely reflecting limited variation in the DFLE-ADL-GBD difference across prefectures.

Supplementary Fig. 1 shows associations with risk factor exposures in 2019. Among the five risk factors examined, only high BMI showed a notable association: higher BMI exposure was negatively correlated with DFLE-AL differences among females (r =  − 0.56, p < 0.001), with a weaker non-significant trend among males (r =  − 0.27, p = 0.068). This indicates that in prefectures with higher obesity prevalence, GBD HALE exceeded MHLW DFLE-AL by a greater margin. Tobacco, high fasting plasma glucose, high blood pressure, and dietary risks showed no significant associations with MHLW-GBD differences for any metric or sex, despite their substantial contributions to disease burden.

Supplementary Fig. 2 presents associations with socioeconomic and healthcare indicators in 2019. For DFLE-AL, several socioeconomic factors showed significant associations. Higher labour force participation was positively correlated with MHLW-GBD differences (males r = 0.31, p = 0.032; females r = 0.33, p = 0.025), indicating that prefectures with higher workforce engagement had relatively higher DFLE-AL compared to GBD HALE. Conversely, higher university graduation rates were negatively correlated with MHLW-GBD differences (males r =  − 0.30, p = 0.038; females r =  − 0.47, p = 0.001), indicating that GBD HALE exceeded MHLW estimates by a greater margin in prefectures with a higher proportion of university-educated residents. Among females, higher annual income was also negatively correlated with DFLE-AL differences (r =  − 0.37, p = 0.010). For LE-SH, associations with socioeconomic indicators were generally weaker and non-significant. For DFLE-ADL, no socioeconomic indicator showed significant associations with the MHLW-GBD difference, consistent with the pattern observed for disease burden and risk factors. Per capita medical expenditure, number of nursing-home facilities, and population age structure showed no consistent associations with any metric.

### Time-varying associations from longitudinal analysis

**Fig. 3:**
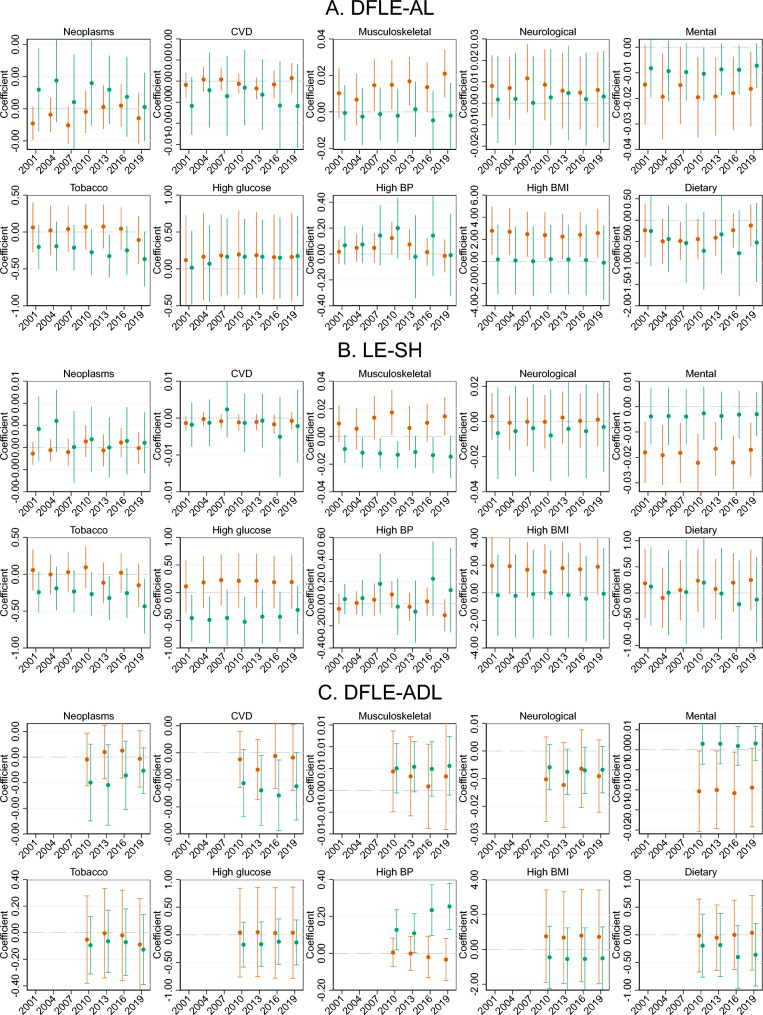
Time-varying coefficients for disease burden indicators and risk factor exposures from fixed-effects panel regression, 2001–2019. Coefficient plots show estimated associations between each covariate and MHLW-GBD 2023 differences at seven time points, derived from fixed-effects models with prefecture and year fixed effects and time-varying coefficients. Panel **A**: DFLE-AL minus GBD 2023 HALE; Panel **B**: LE-SH minus GBD 2023 HALE; Panel **C**: DFLE-ADL minus GBD 2023 HALE. Points indicate coefficient estimates; vertical lines indicate 95% confidence intervals (clustered at prefecture level). Orange: males; green: females. Coefficients represent the change in MHLW-GBD 2023 difference (in years) per unit increase in each covariate. Y-axis scales differ across panels to facilitate visualization of statistical significance (whether confidence intervals cross zero) for each variable. Detailed numerical estimates are provided in Supplementary Table 1

Figure 3 presents time-varying coefficients from fixed-effects panel regression models examining disease burden and risk factor associations with MHLW-GBD differences over 2001–2019 (detailed estimates in Supplementary Table 1). Among males, mental disorder DALYs showed consistently negative associations with the DFLE-AL and LE-SH differences throughout the study period (e.g., DFLE-AL 2019: − 0.016, 95% CI − 0.031 to − 0.002). High BMI exposure showed consistently positive associations with the DFLE-AL difference (e.g., 2019: 2.57, 95% CI 0.37 to 4.77). Musculoskeletal disorder DALYs showed positive associations with DFLE-AL in later years (2019: 0.021, 95% CI 0.008 to 0.034). Among females, tobacco exposure showed negative associations with DFLE-AL and LE-SH differences in recent years (LE-SH 2019: − 0.44, 95% CI − 0.81 to − 0.06), while high fasting plasma glucose showed negative associations with LE-SH in earlier years (2010: − 0.53, 95% CI − 0.97 to − 0.08). For DFLE-ADL, few disease burden or risk factor indicators showed consistent significant associations with the MHLW-GBD difference.

**Fig. 4:**
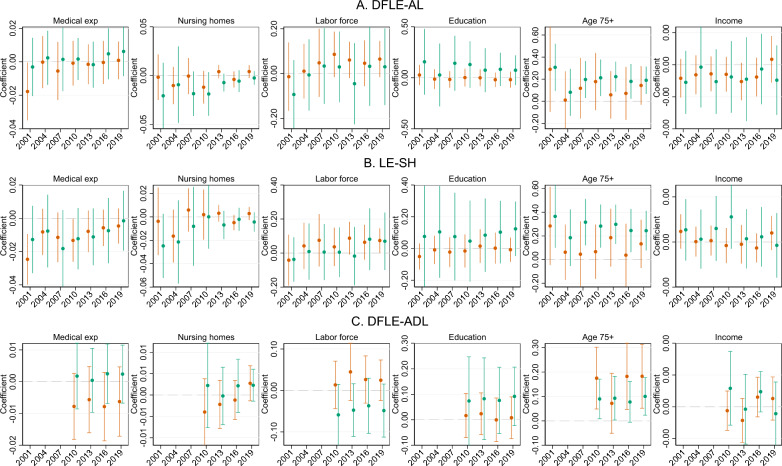
Time-varying coefficients for socioeconomic and healthcare indicators from fixed-effects panel regression, 2001–2019. Coefficient plots show estimated associations between each covariate and MHLW-GBD 2023 differences at seven time points, derived from fixed-effects models with prefecture and year fixed effects and time-varying coefficients. Panel **A**: DFLE-AL minus GBD 2023 HALE; Panel **B**: LE-SH minus GBD 2023 HALE; Panel **C**: DFLE-ADL minus GBD 2023 HALE. Points indicate coefficient estimates; vertical lines indicate 95% confidence intervals (clustered at prefecture level). Orange: males; green: females. Coefficients represent the change in MHLW-GBD 2023 difference (in years) per unit increase in each covariate. Y-axis scales differ across panels to facilitate visualization of statistical significance (whether confidence intervals cross zero) for each variable. Detailed numerical estimates are provided in Supplementary Table 1

Figure 4 shows time-varying coefficients for socioeconomic and healthcare indicators (Supplementary Table 1). The proportion of population aged 75 years and older showed consistently positive associations with DFLE-AL, LE-SH, and DFLE-ADL differences among females throughout the study period (e.g., DFLE-AL 2019: 0.19, 95% CI 0.07 to 0.31; LE-SH 2019: 0.24, 95% CI 0.08 to 0.41). Labour force participation, university graduation rate, annual income, per capita medical expenditure, and number of nursing-home facilities showed no consistent significant patterns across the study period for either sex or any metric.

## Discussion

This study reveals systematic divergence between domestically-used MHLW HALE metrics and the model-based GBD HALE across Japanese prefectures, with concordance patterns that varied markedly by sex and metric type. These findings carry immediate implications for health policy: discordance between HALE metrics is not an error to be reconciled, but a signal that different dimensions of health are being illuminated. The choice of HALE metric shapes how regional health disparities are quantified, which prefectures are identified as priorities, and ultimately how resources are allocated. MHLW metrics and GBD HALE should not be treated as interchangeable indicators, nor should one be dismissed in favour of the other. Rather, they capture complementary dimensions of population health that together provide a more complete picture than either alone.

The most striking pattern was the sex difference in metric concordance. Survey-based metrics (DFLE-AL and LE-SH) correlated strongly with GBD HALE among males but weakly or not at all among females, whereas administrative data-based DFLE-ADL showed consistent concordance regardless of sex. This pattern likely reflects well-documented sex-specific differences in the reporting of poor health, whereby women tend to report worse self-rated health and more functional limitations than men at comparable levels of objectively measured morbidity and mortality [25, 26]. GBD HALE exceeded MHLW estimates in females (by approximately 0.5 years on average) but not in males, suggesting that disease-based modelling may underestimate the health decrements women perceive and report. Women’s health perceptions may be more strongly influenced by symptoms, functional limitations, and psychosocial factors [27] that are incompletely captured in disease-specific disability weights [11, 12]. The substantial caregiving burden borne by Japanese women—who remain primary family caregivers under persistent gender norms—may also contribute to poorer self-reported health beyond what disease burden alone would predict [28]. More broadly, persistent gender inequalities in Japan—including a wide gender pay gap, low representation of women in leadership, and an unequal share of unpaid domestic and care work—translate into chronic stressors and caregiving burdens disproportionately borne by women, which have been linked to poorer self-rated and psychological health among Japanese women [28–30]. Because such gendered structural factors are not directly represented in the GBD disease-and-disability framework, they may help explain why survey-based MHLW metrics diverge from GBD HALE more strongly among females. Relying solely on GBD HALE for regional comparisons may thus systematically mischaracterise health disparities among women. In contrast, DFLE-ADL—derived from objective certification of long-term care needs based on standardised assessment criteria rather than self-reported perceptions—showed consistent concordance with GBD HALE in both sexes.

The strong correlation between DFLE-AL and LE-SH (r = 0.66–0.89 across years and sexes) indicates that functional limitations and subjective health perceptions are closely related but not identical constructs. Both survey-based metrics showed similar patterns of divergence from GBD HALE, suggesting that the gap between self-reported and model-based health assessment is consistent regardless of whether the survey captures activity limitations or general health perceptions. This consistency strengthens confidence that the observed divergence reflects genuine differences between measurement approaches rather than idiosyncrasies of a particular survey question [31].

Our analyses employed two complementary approaches—cross-sectional correlations and longitudinal panel regression with time-varying coefficients—which yielded partially divergent findings that merit careful interpretation. In cross-sectional analysis using 2019 data, cardiovascular disease burden showed strong correlations with divergence (DFLE-AL: r = 0.52 for males, r = 0.41 for females), and socioeconomic indicators including university graduation rate, annual income, and labour force participation were significantly associated with metric divergence. However, when we applied fixed-effects panel regression controlling for time-invariant prefecture characteristics and shared temporal trends, these associations did not persist. This means that while prefectures with higher cardiovascular burden or specific socioeconomic profiles tend to show greater metric divergence at any given time point, changes in these factors within a prefecture over time were not associated with corresponding changes in divergence. One interpretation is that both disease burden and metric divergence are shaped by stable underlying prefecture characteristics—such as regional healthcare culture, health awareness, or reporting norms—that confound the cross-sectional association. Alternatively, within-prefecture variation over the 19-year study period may have been insufficient to detect temporal associations. These cross-sectional patterns nonetheless have practical relevance: they indicate that the choice of HALE metric may have differential implications for identifying health priorities across socioeconomically diverse regions, even if the factors do not causally drive divergence.

In contrast, several findings emerged specifically from the longitudinal analysis. The negative association between mental disorder burden and metric divergence among males suggests that survey-based metrics may be more sensitive to the functional and psychological impacts of mental disorders on daily life than GBD’s disease-specific modelling [32, 33]. Mental health conditions may affect self-reported activity limitations and subjective health in ways that disability weights—derived from general population valuations of hypothetical health states—do not fully capture [12, 34]. Conversely, the positive associations observed for musculoskeletal disorders and high BMI among males suggest that individuals may adapt to these chronic conditions and not report them as functional limitations, even when epidemiological models assign substantial disability [35, 36]. This pattern of adaptation may explain why MHLW estimates exceeded GBD HALE by a greater margin in prefectures with higher musculoskeletal burden or obesity prevalence.

The most consistent finding from longitudinal analysis was the positive association between population ageing and metric divergence among females. This pattern likely reflects response shift, whereby older adults adapt their health expectations and maintain favourable self-rated health even as disease burden increases [37]. Such adaptation would lead to higher MHLW estimates relative to GBD HALE in demographically older prefectures. This finding has direct relevance for Japan’s rapidly ageing society and suggests that demographic structure systematically influences how survey-based HALE metrics compare with GBD HALE.

Rather than seeking convergence across metrics, divergent patterns can be used to tailor subnational strategies to the specific health dimensions most salient in each context. These findings carry strategic implications for national health planning. Health Japan 21 has adopted MHLW’s DFLE-AL as a primary policy target [38], while international comparisons and the SDG framework draw on standardised estimates such as those produced by GBD and WHO [1, 13]. Our results caution against treating these metrics as equivalent. If policy decisions are based exclusively on GBD HALE, prefectures where residents experience substantial functional limitations or poor subjective health—particularly among women—may be overlooked. If based exclusively on MHLW metrics, comparability with international benchmarks is compromised. A multi-metric approach is warranted: GBD HALE for standardised international comparison, survey-based MHLW metrics for capturing self-reported health dimensions that matter to individuals but may escape disease-focused quantification, and administrative data-based DFLE-ADL for objective assessment of functional independence [7]. Discordance between metrics should itself become a policy signal, prompting investigation into why populations report health experiences that diverge from model predictions.

For the GBD enterprise, our findings highlight the value of subnational validation against locally collected health data. The systematic patterns of divergence documented here—by sex and demographic structure—are not random noise but structured signals that could inform model refinement. Disability weights derived from broader population surveys, including non-Western samples, might reduce context-specific bias [39, 40]. For national statistical agencies, our results underscore the continued importance of population health surveys that directly assess functional limitations and subjective health, even as model-based approaches grow more sophisticated. The tension between these measurement traditions is productive: it reveals what each approach captures and what each misses.

### Limitations

Several limitations warrant consideration. First, the ecological design precludes causal inference and individual-level interpretation; associations observed at the prefecture level may not hold for individuals (ecological fallacy), and prefecture-level covariates such as disease burden reflect population averages rather than the health status of individuals responding to surveys. Second, linear interpolation and extrapolation for years with missing socioeconomic data introduced measurement error, potentially attenuating associations. Third, we could not examine all potential confounders, including healthcare quality indicators, cultural factors influencing health reporting, and urban–rural differences that may vary systematically across prefectures. Fourth, our analysis focused on Japan; generalisability to other high-income countries where GBD and national HALE metrics may diverge requires further investigation, though the conceptual tensions we identify likely apply broadly. Fifth, the four HALE metrics rely on different underlying life tables and mortality inputs, so the observed MHLW–GBD differences reflect both differences in mortality inputs and differences in how disability is operationalised. The MHLW indicators are based on Japanese national or prefectural life tables derived directly from vital registration and census data, whereas GBD HALE is computed from GBD-modelled, location-specific mortality estimates produced within the GBD statistical modelling pipeline. Although both ultimately reflect Japanese mortality experience, differences in data processing, smoothing, modelling approach, and geographic granularity may contribute to the observed gaps. Formal decomposition of the mortality- and disability-related contributions to these differences would be an important topic for future methodological work. Finally, we examined only three of multiple possible HALE definitions used in Japan; other metrics based on specific functional domains may show different patterns of concordance with GBD estimates.

## Conclusions

The divergence between MHLW and GBD HALE metrics is neither a measurement artefact to be resolved nor an inconvenience to be ignored—it is an informative signal about what different approaches to health quantification capture and miss. Our findings demonstrate that neither MHLW metrics nor GBD HALE alone provides a complete representation of population health across Japanese prefectures. Policymakers should resist the temptation to select a single ‘correct’ metric and instead embrace a multi-metric framework that leverages the strengths of each approach: GBD HALE for standardised international comparison and epidemiological anchoring, survey-based MHLW metrics for capturing self-reported functional limitations and subjective health that matter for individuals’ daily lives, and administrative data-based DFLE-ADL for objective assessment of care needs that shows consistent concordance with GBD HALE across both sexes. Discordance between metrics, particularly the systematic sex differences in survey-based metrics documented here, should prompt deeper investigation rather than dismissal. As Japan and other nations pursue ambitious targets to extend HALE, similar tensions between national data systems and global models are likely to emerge elsewhere, underscoring the broader relevance of this case study; the measurement choices we make will shape which populations we see, which we overlook, and ultimately whose health we prioritise.

## Supplementary Information

Supplementary material 1.

## Data Availability

All data used in this study are publicly available. Prefecture-level healthy life expectancy estimates from Japan’s Ministry of Health, Labour and Welfare are available from the Healthy Life Expectancy dashboard (https://toukei.umin.jp/kenkoujyumyou/). Global Burden of Disease 2023 estimates are available from the GBD Results Tool (https://vizhub.healthdata.org/gbd-results/). Socioeconomic indicators are available from the Japanese government statistics portal e-Stat (https://www.e-stat.go.jp/). The analytical code and compiled dataset are available from the corresponding author upon reasonable request.

## Abbreviations

CI: Confidence interval
DALYs: Disability-adjusted life-years
DFLE-ADL: Disability-free life expectancy based on activities of daily living
DFLE-AL: Disability-free life expectancy based on activity limitation
GBD: Global burden of disease
HALE: Healthy life expectancy
LE-SH: Life expectancy in subjective health
LTCI: Long-term care insurance
MHLW: Ministry of health, Labour and welfare
SDGs: Sustainable development goals
SEV: Summary exposure value
YLDs: Years lived with disability

## Declarations

### Patient and public involvement

Patients and/or the public were not involved in the design, or conduct, or reporting, or dissemination plans of this research.
