# Supplementary material for: Why healthy life expectancy estimates diverge: insights from survey-based, administrative, and model-based metrics across Japanese prefectures, 2001–2019: a comparative ecological study
Source: Popul Health Metr. 2026 Jun 10;24:50. doi: 10.1186/s12963-026-00488-z (PMC13474879; doi:10.1186/s12963-026-00488-z)
Supplement: Supplementary file 1 [file 12963_2026_488_MOESM1_ESM.docx]

**Supplementary Material**

**Table of Contents**

Supplementary Text: Detailed description of covariates

Supplementary Figure 1: Cross-sectional correlations between MHLW-GBD 2023 differences and risk factor exposures, 2019

Supplementary Figure 2: Cross-sectional correlations between MHLW-GBD 2023 differences and socioeconomic and healthcare indicators, 2019

Supplementary Table 1: Association between prefecture-level factors and differences between MHLW HALE measures and GBD HALE

**Supplementary Text**: **Detailed description of covariates**

**Rationale for covariate selection**

To examine factors associated with divergence between Ministry of Health, Labour and Welfare (MHLW) and Global Burden of Disease (GBD) healthy life expectancy (HALE) metrics, we assembled covariates from three domains: disease burden indicators, risk factor exposures, and socioeconomic and healthcare indicators. This selection was guided by the hypothesis that different types of factors may differentially influence survey-based versus model-based health metrics, thereby contributing to divergence between measurement approaches. All covariates were extracted for the seven analysis years: 2001, 2004, 2007, 2010, 2013, 2016, and 2019.

**Disease burden indicators**

We obtained age-standardised disability-adjusted life-years (DALYs) per 100,000 population from GBD 2023 for the five leading Level 2 causes in Japan: neoplasms, cardiovascular diseases, musculoskeletal disorders, neurological disorders, and mental disorders.[1] GBD HALE incorporates disease burden directly through its estimation process, weighting health states by disease-specific disability weights, whereas MHLW metrics capture functional limitations and subjective health perceptions that are not explicitly linked to disease categories. Detailed definitions and corresponding International Classification of Diseases, 10th Revision (ICD-10) codes for each cause are available in GBD documentation.[1]

**Risk factor exposures**

We obtained age-standardised summary exposure values (SEV) from GBD 2023 for five major Level 2 risk factors in Japan: tobacco use, high fasting plasma glucose, high systolic blood pressure, high body-mass index (BMI), and dietary risks.[1] SEV quantifies population-level risk exposure on a 0–100 scale, representing the proportion of risk-weighted exposure relative to the theoretical maximum.[2] Risk factors represent upstream determinants that influence GBD HALE through disease incidence pathways, while potentially affecting self-reported health through different mechanisms such as health awareness or lifestyle factors. Detailed definitions for each risk factor are available in GBD documentation.[2]

**Socioeconomic and healthcare indicators**

Socioeconomic and healthcare indicators were included because these factors may shape health perceptions independently of disease burden, potentially influencing MHLW metrics more than model-based GBD HALE. These indicators serve as proxies for the social and cultural environment that may influence health literacy and reporting thresholds among survey respondents. We selected indicators representing key social determinants across multiple domains—healthcare resources, economic status, education, employment, and demographic structure—that have established associations with self-rated health and are monitored in Japanese health policy. We prioritised indicators with prefecture-level and sex-stratified data available from official government statistics. Prefecture-level data were compiled from Japanese government sources via e-Stat.[3]

Healthcare indicators included:

- Per capita medical expenditure (thousand yen): Total medical expenditure including public funding, insurance premiums, and patients' out-of-pocket payments, divided by mid-year population, from the National Medical Care Expenditure survey.[4]
- Number of nursing-home facilities (per 100,000 population aged ≥65 years): Comprising nursing homes for older adults, long-term care welfare facilities, low-cost homes for older adults, and fee-based homes for older adults, from the System of Social and Demographic Statistics.[5]

Socioeconomic indicators comprised:

- Labour force participation rate: Percentage of the population aged 15 years and over in the labour force (employed plus unemployed), obtained from the System of Social and Demographic Statistics.[5]
- University graduation rate: Proportion of individuals who have graduated from university or graduate school among the population aged 15 years and over, from the Population Census.[6]
- Annual income (thousand yen): Sum of monthly contractual cash earnings over 12 months and annual bonuses, from the Basic Survey on Wage Structure.[7]
- Proportion of population aged 75 years and older: From the System of Social and Demographic Statistics.[5]

**Data availability and interpolation**

Data for disease burden indicators, risk factor exposures, nursing-home facilities, annual income, and proportion aged ≥75 years were available for all seven analysis years (2001, 2004, 2007, 2010, 2013, 2016, and 2019). The following three indicators were not uniformly available for all analysis years:

- Per capita medical expenditure: available triennially (2002, 2005, 2008, 2011, 2014, 2016, 2019)
- Labour force participation rate by sex: available quinquennially (2000, 2005, 2010, 2015, 2020)
- University graduation rate: available from the quinquennial Population Census (2002, 2007, 2012, 2017)

For these three indicators, we applied linear interpolation between adjacent data points for years without observed data, or linear extrapolation when the target year fell outside available data, assuming constant rate of change.

**References**

1.	Institute for Health Metrics and Evaluation. VizHub - GBD Results. 2025. <https://vizhub.healthdata.org/gbd-results/> (accessed December 24, 2025).

2.	GBD 2023 Disease and Injury and Risk Factor Collaborators. Burden of 375 diseases and injuries, risk-attributable burden of 88 risk factors, and healthy life expectancy in 204 countries and territories, including 660 subnational locations, 1990-2023: a systematic analysis for the Global Burden of Disease Study 2023. *Lancet* 2025; **406**(10513): 1873-922.

3.	e-Stat. Statistics of Japan. <https://www.e-stat.go.jp/en> (accessed December 24, 2025).

4.	e-Stat. National Medical Care Expenditure [Japanese]. <https://www.e-stat.go.jp/statistics/00450032> (accessed December 24, 2025).

5.	e-Stat. System of Social and Demographic Statistics [Japanese]. <https://www.e-stat.go.jp/statistics/00200502> (accessed December 24, 2025).

6.	e-Stat. Population Census [Japanese]. <https://www.e-stat.go.jp/statistics/00200521> (accessed December 24, 2025).

7.	e-Stat. Basic Survey on Wage Structure [Japanese]. <https://www.e-stat.go.jp/statistics/00450091> (accessed December 24, 2025).

**Supplementary Figure 1. Cross-sectional correlations between MHLW-GBD 2023 differences and risk factor exposures, 2019.** Scatter plots show the relationship between the difference in HALE estimates (MHLW minus GBD 2023; y-axis) and age-standardised summary exposure values (SEV) from GBD 2023 (x-axis) for five major risk factors. First row: DFLE-AL minus GBD 2023 HALE; second row: LE-SH minus GBD 2023 HALE; third row: DFLE-ADL minus GBD 2023 HALE. Risk factors include tobacco use, high fasting plasma glucose, high blood pressure, high body-mass index (BMI), and dietary risks. Pearson correlation coefficients and p-values are shown for males (orange) and females (green).

**Supplementary Figure 2. Cross-sectional correlations between MHLW-GBD 2023 differences and socioeconomic and healthcare indicators, 2019.** Scatter plots show the relationship between the difference in HALE estimates (MHLW minus GBD 2023; y-axis) and six socioeconomic and healthcare indicators (x-axis). First row: DFLE-AL minus GBD 2023 HALE; second row: LE-SH minus GBD 2023 HALE; third row: DFLE-ADL minus GBD 2023 HALE. Indicators include per capita medical care expenditure, nursing home availability per 100,000 population aged ≥65 years, labour force participation rate, university graduation rate, proportion of population aged ≥75 years, and annual income. Pearson correlation coefficients and p-values are shown for males (M; orange) and females (F; green).

**Supplementary Table 1.** Association between prefecture-level factors and difference in HALE estimates (MHLW minus GBD 2023): panel regression coefficients (95% CI)

**Panel A. DFLE-AL**

| **Variable** | **Sex** | **2001** | **2004** | **2007** | **2010** | **2013** | **2016** | **2019** |
| --- | --- | --- | --- | --- | --- | --- | --- | --- |
| ***Disease burden indicators*** |  |  |  |  |  |  |  |  |
| Cardiovascular diseases | Male | -0.000 (-0.002 to 0.001) | 0.000 (-0.001 to 0.001) | 0.000 (-0.001 to 0.001) | -0.000 (-0.001 to 0.001) | -0.001 (-0.002 to 0.000) | -0.000 (-0.002 to 0.001) | 0.000 (-0.001 to 0.002) |
|  | Female | -0.002 (-0.005 to 0.000) | -0.001 (-0.004 to 0.003) | -0.001 (-0.005 to 0.002) | -0.001 (-0.004 to 0.003) | -0.001 (-0.005 to 0.002) | -0.002 (-0.006 to 0.002) | -0.002 (-0.006 to 0.002) |
| Neoplasms | Male | -0.001 (-0.002 to 0.000) | -0.000 (-0.001 to 0.001) | -0.001 (-0.002 to 0.000) | -0.000 (-0.002 to 0.001) | 0.000 (-0.001 to 0.001) | 0.000 (-0.001 to 0.002) | -0.001 (-0.002 to 0.001) |
|  | Female | 0.001 (-0.001 to 0.004) | 0.002 (-0.001 to 0.004) | 0.000 (-0.003 to 0.003) | 0.002 (-0.001 to 0.004) | 0.001 (-0.001 to 0.003) | 0.001 (-0.002 to 0.003) | 0.000 (-0.002 to 0.002) |
| Musculoskeletal disorders | Male | 0.010 (-0.003 to 0.024) | 0.007 (-0.007 to 0.021) | **0.015 (0.000 to 0.029)** | **0.015 (0.001 to 0.028)** | **0.017 (0.004 to 0.030)** | 0.014 (-0.000 to 0.027) | **0.021 (0.008 to 0.034)** |
|  | Female | -0.001 (-0.016 to 0.015) | -0.003 (-0.018 to 0.013) | -0.001 (-0.016 to 0.013) | -0.002 (-0.017 to 0.013) | 0.001 (-0.014 to 0.017) | -0.005 (-0.021 to 0.011) | -0.002 (-0.020 to 0.016) |
| Neurological disorders | Male | 0.008 (-0.006 to 0.022) | 0.007 (-0.008 to 0.022) | 0.012 (-0.004 to 0.027) | 0.009 (-0.008 to 0.025) | 0.006 (-0.012 to 0.024) | 0.005 (-0.012 to 0.022) | 0.006 (-0.011 to 0.024) |
|  | Female | 0.002 (-0.018 to 0.022) | 0.002 (-0.019 to 0.023) | 0.000 (-0.022 to 0.022) | 0.003 (-0.020 to 0.025) | 0.005 (-0.017 to 0.027) | 0.002 (-0.020 to 0.024) | 0.003 (-0.018 to 0.024) |
| Mental disorders | Male | -0.015 (-0.030 to 0.001) | **-0.019 (-0.036 to -0.003)** | -0.015 (-0.030 to 0.000) | **-0.020 (-0.035 to -0.004)** | **-0.019 (-0.034 to -0.004)** | **-0.018 (-0.032 to -0.004)** | **-0.016 (-0.031 to -0.002)** |
|  | Female | -0.008 (-0.020 to 0.003) | -0.009 (-0.021 to 0.003) | -0.010 (-0.021 to 0.002) | -0.010 (-0.021 to 0.000) | -0.009 (-0.019 to 0.001) | -0.009 (-0.018 to 0.001) | -0.007 (-0.016 to 0.002) |
| ***Risk factor exposures*** |  |  |  |  |  |  |  |  |
| High blood pressure | Male | 0.018 (-0.076 to 0.111) | 0.047 (-0.060 to 0.155) | 0.047 (-0.067 to 0.162) | 0.123 (-0.003 to 0.248) | 0.073 (-0.044 to 0.191) | 0.014 (-0.093 to 0.122) | -0.014 (-0.140 to 0.112) |
|  | Female | 0.067 (-0.081 to 0.215) | 0.073 (-0.072 to 0.218) | 0.143 (-0.090 to 0.375) | 0.200 (-0.034 to 0.433) | -0.021 (-0.341 to 0.298) | 0.142 (-0.170 to 0.454) | -0.009 (-0.325 to 0.307) |
| High fasting plasma glucose | Male | 0.119 (-0.489 to 0.727) | 0.165 (-0.427 to 0.756) | 0.182 (-0.375 to 0.739) | 0.195 (-0.399 to 0.790) | 0.185 (-0.394 to 0.765) | 0.159 (-0.418 to 0.736) | 0.162 (-0.434 to 0.759) |
|  | Female | 0.017 (-0.484 to 0.518) | 0.070 (-0.447 to 0.587) | 0.165 (-0.358 to 0.688) | 0.164 (-0.351 to 0.679) | 0.165 (-0.339 to 0.669) | 0.151 (-0.398 to 0.700) | 0.174 (-0.369 to 0.718) |
| High BMI | Male | **2.780 (0.616 to 4.944)** | **2.689 (0.552 to 4.826)** | **2.471 (0.438 to 4.504)** | **2.382 (0.327 to 4.437)** | **2.248 (0.214 to 4.281)** | **2.419 (0.403 to 4.436)** | **2.570 (0.366 to 4.774)** |
|  | Female | 0.194 (-2.932 to 3.321) | 0.089 (-2.969 to 3.147) | 0.023 (-3.112 to 3.159) | 0.211 (-2.861 to 3.283) | 0.190 (-2.957 to 3.337) | 0.134 (-3.062 to 3.329) | -0.112 (-3.457 to 3.233) |
| Tobacco | Male | 0.062 (-0.275 to 0.398) | 0.021 (-0.317 to 0.359) | 0.041 (-0.268 to 0.350) | 0.069 (-0.237 to 0.374) | 0.074 (-0.222 to 0.371) | 0.044 (-0.246 to 0.333) | -0.108 (-0.432 to 0.217) |
|  | Female | -0.203 (-0.502 to 0.097) | -0.193 (-0.526 to 0.139) | -0.212 (-0.521 to 0.096) | -0.274 (-0.586 to 0.039) | **-0.327 (-0.617 to -0.036)** | -0.250 (-0.582 to 0.083) | -0.367 (-0.738 to 0.005) |
| Dietary risk | Male | -0.236 (-0.863 to 0.391) | -0.493 (-1.023 to 0.038) | **-0.484 (-0.928 to -0.040)** | -0.441 (-0.913 to 0.031) | -0.409 (-0.836 to 0.017) | -0.235 (-0.641 to 0.172) | -0.123 (-0.607 to 0.361) |
|  | Female | -0.252 (-1.069 to 0.565) | -0.439 (-1.212 to 0.333) | -0.535 (-1.460 to 0.391) | -0.714 (-1.619 to 0.191) | -0.330 (-1.247 to 0.586) | -0.771 (-1.778 to 0.235) | -0.521 (-1.444 to 0.401) |
| ***Socioeconomic and healthcare indicators*** |  |  |  |  |  |  |  |  |
| Per capita medical exp | Male | **-0.018 (-0.035 to -0.001)** | -0.000 (-0.016 to 0.015) | -0.005 (-0.023 to 0.012) | -0.001 (-0.014 to 0.012) | -0.001 (-0.013 to 0.010) | -0.000 (-0.013 to 0.012) | 0.001 (-0.010 to 0.012) |
|  | Female | -0.003 (-0.020 to 0.014) | 0.002 (-0.014 to 0.018) | 0.001 (-0.017 to 0.020) | 0.002 (-0.011 to 0.014) | -0.002 (-0.015 to 0.012) | 0.005 (-0.010 to 0.020) | 0.006 (-0.009 to 0.021) |
| Nursing homes per 100k | Male | -0.002 (-0.025 to 0.022) | -0.010 (-0.029 to 0.009) | -0.001 (-0.019 to 0.018) | -0.012 (-0.029 to 0.005) | 0.004 (-0.003 to 0.011) | -0.004 (-0.012 to 0.004) | 0.004 (-0.002 to 0.011) |
|  | Female | -0.021 (-0.055 to 0.013) | -0.009 (-0.049 to 0.030) | -0.019 (-0.041 to 0.004) | -0.019 (-0.041 to 0.003) | -0.007 (-0.016 to 0.002) | -0.006 (-0.017 to 0.005) | -0.002 (-0.009 to 0.005) |
| Labor force ratio (%) | Male | -0.014 (-0.167 to 0.139) | 0.011 (-0.112 to 0.133) | 0.048 (-0.104 to 0.200) | 0.086 (-0.016 to 0.187) | 0.060 (-0.020 to 0.141) | 0.046 (-0.057 to 0.148) | 0.065 (-0.016 to 0.146) |
|  | Female | -0.094 (-0.247 to 0.060) | -0.006 (-0.166 to 0.153) | 0.033 (-0.135 to 0.202) | 0.030 (-0.128 to 0.187) | -0.045 (-0.227 to 0.136) | 0.032 (-0.144 to 0.209) | 0.030 (-0.141 to 0.201) |
| University grad ratio | Male | 0.027 (-0.072 to 0.126) | -0.013 (-0.109 to 0.084) | -0.017 (-0.098 to 0.065) | 0.002 (-0.085 to 0.089) | -0.000 (-0.083 to 0.083) | -0.019 (-0.090 to 0.052) | -0.019 (-0.097 to 0.060) |
|  | Female | 0.157 (-0.165 to 0.479) | 0.027 (-0.280 to 0.335) | 0.142 (-0.126 to 0.411) | 0.130 (-0.098 to 0.358) | 0.075 (-0.140 to 0.289) | 0.083 (-0.080 to 0.245) | 0.074 (-0.071 to 0.220) |
| Pop aged 75+ (%) | Male | 0.290 (-0.095 to 0.674) | 0.011 (-0.249 to 0.271) | 0.118 (-0.156 to 0.392) | 0.178 (-0.080 to 0.436) | 0.059 (-0.156 to 0.273) | 0.072 (-0.168 to 0.311) | 0.144 (-0.031 to 0.318) |
|  | Female | **0.307 (0.094 to 0.521)** | 0.082 (-0.130 to 0.294) | **0.197 (0.031 to 0.364)** | **0.213 (0.048 to 0.378)** | **0.223 (0.088 to 0.358)** | **0.180 (0.023 to 0.337)** | **0.190 (0.067 to 0.313)** |
| Annual income | Male | -0.000 (-0.001 to 0.000) | -0.000 (-0.001 to 0.000) | -0.000 (-0.001 to 0.000) | -0.000 (-0.001 to 0.000) | -0.001 (-0.001 to 0.000) | -0.000 (-0.001 to 0.000) | 0.000 (-0.001 to 0.001) |
|  | Female | -0.001 (-0.002 to 0.000) | -0.000 (-0.001 to 0.001) | -0.001 (-0.002 to 0.000) | -0.000 (-0.001 to 0.001) | -0.000 (-0.002 to 0.001) | -0.000 (-0.001 to 0.001) | -0.000 (-0.002 to 0.001) |

**Panel B. LE-SH**

| **Variable** | **Sex** | **2001** | **2004** | **2007** | **2010** | **2013** | **2016** | **2019** |
| --- | --- | --- | --- | --- | --- | --- | --- | --- |
| ***Disease burden indicators*** |  |  |  |  |  |  |  |  |
| Cardiovascular diseases | Male | -0.001 (-0.002 to 0.000) | -0.000 (-0.001 to 0.001) | -0.000 (-0.001 to 0.001) | -0.001 (-0.002 to 0.001) | -0.001 (-0.002 to 0.001) | -0.001 (-0.002 to 0.000) | -0.000 (-0.002 to 0.001) |
|  | Female | -0.001 (-0.004 to 0.002) | -0.001 (-0.004 to 0.003) | 0.001 (-0.003 to 0.005) | -0.001 (-0.005 to 0.003) | -0.000 (-0.004 to 0.003) | -0.003 (-0.008 to 0.003) | -0.001 (-0.006 to 0.004) |
| Neoplasms | Male | -0.001 (-0.002 to 0.000) | -0.000 (-0.001 to 0.001) | -0.000 (-0.002 to 0.001) | 0.001 (-0.001 to 0.002) | -0.000 (-0.002 to 0.001) | 0.000 (-0.001 to 0.002) | -0.000 (-0.001 to 0.001) |
|  | Female | 0.002 (-0.001 to 0.005) | 0.002 (-0.000 to 0.005) | 0.000 (-0.003 to 0.003) | 0.001 (-0.002 to 0.004) | 0.000 (-0.003 to 0.003) | 0.001 (-0.002 to 0.004) | 0.000 (-0.002 to 0.003) |
| Musculoskeletal disorders | Male | 0.009 (-0.004 to 0.023) | 0.006 (-0.009 to 0.021) | 0.014 (-0.002 to 0.029) | **0.017 (0.001 to 0.033)** | 0.006 (-0.010 to 0.022) | 0.010 (-0.004 to 0.024) | **0.015 (0.001 to 0.028)** |
|  | Female | -0.009 (-0.019 to 0.001) | **-0.012 (-0.023 to -0.001)** | **-0.012 (-0.023 to -0.001)** | **-0.013 (-0.023 to -0.003)** | -0.011 (-0.023 to 0.000) | **-0.014 (-0.027 to -0.000)** | -0.015 (-0.030 to 0.001) |
| Neurological disorders | Male | 0.003 (-0.010 to 0.016) | -0.001 (-0.016 to 0.015) | -0.000 (-0.014 to 0.014) | -0.000 (-0.015 to 0.015) | 0.002 (-0.013 to 0.017) | 0.000 (-0.015 to 0.016) | 0.001 (-0.014 to 0.016) |
|  | Female | -0.007 (-0.033 to 0.019) | -0.005 (-0.031 to 0.020) | -0.004 (-0.029 to 0.021) | -0.008 (-0.034 to 0.018) | -0.004 (-0.031 to 0.023) | -0.006 (-0.032 to 0.021) | -0.003 (-0.029 to 0.022) |
| Mental disorders | Male | **-0.018 (-0.030 to -0.006)** | **-0.019 (-0.031 to -0.007)** | **-0.018 (-0.030 to -0.006)** | **-0.022 (-0.034 to -0.011)** | **-0.017 (-0.027 to -0.006)** | **-0.022 (-0.033 to -0.011)** | **-0.017 (-0.028 to -0.006)** |
|  | Female | -0.004 (-0.015 to 0.007) | -0.004 (-0.015 to 0.008) | -0.004 (-0.015 to 0.007) | -0.003 (-0.013 to 0.008) | -0.004 (-0.013 to 0.006) | -0.003 (-0.013 to 0.006) | -0.003 (-0.012 to 0.006) |
| ***Risk factor exposures*** |  |  |  |  |  |  |  |  |
| High blood pressure | Male | -0.046 (-0.181 to 0.089) | 0.007 (-0.098 to 0.113) | 0.036 (-0.109 to 0.181) | 0.084 (-0.036 to 0.204) | -0.028 (-0.157 to 0.102) | 0.021 (-0.105 to 0.147) | -0.104 (-0.250 to 0.041) |
|  | Female | 0.041 (-0.094 to 0.177) | 0.050 (-0.112 to 0.213) | 0.180 (-0.089 to 0.449) | -0.027 (-0.283 to 0.229) | -0.071 (-0.352 to 0.209) | 0.226 (-0.109 to 0.562) | 0.123 (-0.260 to 0.506) |
| High fasting plasma glucose | Male | 0.114 (-0.361 to 0.590) | 0.185 (-0.282 to 0.652) | 0.227 (-0.233 to 0.687) | 0.215 (-0.282 to 0.713) | 0.215 (-0.268 to 0.697) | 0.189 (-0.284 to 0.661) | 0.193 (-0.291 to 0.678) |
|  | Female | **-0.459 (-0.883 to -0.036)** | **-0.492 (-0.912 to -0.072)** | -0.456 (-0.965 to 0.052) | **-0.526 (-0.973 to -0.080)** | -0.433 (-0.921 to 0.055) | -0.435 (-0.889 to 0.019) | -0.313 (-0.749 to 0.124) |
| High BMI | Male | 1.960 (-0.100 to 4.020) | 1.934 (-0.105 to 3.972) | 1.674 (-0.339 to 3.686) | 1.531 (-0.474 to 3.537) | 1.786 (-0.159 to 3.730) | 1.706 (-0.217 to 3.629) | 1.883 (-0.133 to 3.899) |
|  | Female | -0.176 (-3.121 to 2.768) | -0.215 (-3.228 to 2.798) | -0.091 (-3.291 to 3.110) | -0.017 (-3.115 to 3.081) | -0.168 (-3.248 to 2.913) | -0.432 (-3.511 to 2.648) | -0.070 (-3.374 to 3.234) |
| Tobacco | Male | 0.056 (-0.224 to 0.335) | -0.003 (-0.276 to 0.271) | 0.027 (-0.246 to 0.301) | 0.094 (-0.188 to 0.375) | -0.116 (-0.392 to 0.160) | 0.019 (-0.252 to 0.290) | -0.149 (-0.445 to 0.146) |
|  | Female | -0.243 (-0.522 to 0.036) | -0.192 (-0.486 to 0.103) | -0.234 (-0.521 to 0.054) | -0.269 (-0.567 to 0.028) | **-0.323 (-0.620 to -0.025)** | -0.255 (-0.594 to 0.083) | **-0.435 (-0.808 to -0.062)** |
| Dietary risk | Male | 0.186 (-0.475 to 0.847) | -0.092 (-0.665 to 0.481) | 0.058 (-0.522 to 0.638) | 0.236 (-0.364 to 0.836) | 0.078 (-0.498 to 0.653) | 0.198 (-0.365 to 0.761) | 0.249 (-0.331 to 0.829) |
|  | Female | 0.124 (-0.626 to 0.875) | 0.006 (-0.804 to 0.816) | 0.018 (-0.976 to 1.013) | 0.200 (-0.667 to 1.066) | -0.010 (-0.884 to 0.864) | -0.214 (-1.052 to 0.624) | -0.126 (-0.936 to 0.684) |
| ***Socioeconomic and healthcare indicators*** |  |  |  |  |  |  |  |  |
| Per capita medical exp | Male | **-0.025 (-0.040 to -0.009)** | -0.008 (-0.022 to 0.006) | -0.011 (-0.026 to 0.003) | **-0.013 (-0.026 to -0.000)** | -0.008 (-0.020 to 0.005) | -0.006 (-0.017 to 0.005) | -0.005 (-0.015 to 0.006) |
|  | Female | -0.013 (-0.033 to 0.007) | -0.008 (-0.030 to 0.014) | -0.018 (-0.041 to 0.005) | -0.012 (-0.031 to 0.006) | -0.011 (-0.028 to 0.006) | -0.007 (-0.027 to 0.012) | -0.002 (-0.020 to 0.016) |
| Nursing homes per 100k | Male | -0.004 (-0.033 to 0.025) | -0.016 (-0.039 to 0.006) | 0.006 (-0.013 to 0.024) | 0.002 (-0.019 to 0.023) | 0.003 (-0.004 to 0.010) | -0.005 (-0.011 to 0.002) | 0.003 (-0.003 to 0.008) |
|  | Female | -0.025 (-0.052 to 0.003) | -0.021 (-0.057 to 0.014) | -0.008 (-0.042 to 0.026) | 0.000 (-0.027 to 0.027) | -0.007 (-0.019 to 0.005) | -0.002 (-0.012 to 0.008) | -0.004 (-0.013 to 0.004) |
| Labor force ratio (%) | Male | -0.042 (-0.192 to 0.108) | 0.042 (-0.094 to 0.178) | 0.075 (-0.079 to 0.229) | 0.036 (-0.077 to 0.149) | 0.087 (-0.009 to 0.183) | 0.064 (-0.039 to 0.166) | **0.073 (0.002 to 0.145)** |
|  | Female | -0.037 (-0.168 to 0.094) | 0.009 (-0.157 to 0.174) | 0.006 (-0.159 to 0.170) | 0.005 (-0.141 to 0.151) | -0.018 (-0.189 to 0.154) | 0.081 (-0.100 to 0.263) | 0.070 (-0.099 to 0.238) |
| University grad ratio | Male | -0.051 (-0.133 to 0.032) | -0.009 (-0.098 to 0.080) | -0.023 (-0.117 to 0.071) | -0.016 (-0.120 to 0.088) | 0.015 (-0.091 to 0.121) | 0.002 (-0.096 to 0.100) | -0.008 (-0.084 to 0.068) |
|  | Female | 0.076 (-0.243 to 0.395) | 0.104 (-0.185 to 0.394) | 0.075 (-0.201 to 0.351) | 0.046 (-0.209 to 0.301) | 0.084 (-0.147 to 0.315) | 0.103 (-0.094 to 0.300) | 0.124 (-0.048 to 0.295) |
| Pop aged 75+ (%) | Male | 0.284 (-0.046 to 0.615) | 0.063 (-0.171 to 0.296) | 0.046 (-0.232 to 0.324) | 0.067 (-0.163 to 0.297) | 0.185 (-0.060 to 0.430) | 0.038 (-0.228 to 0.303) | 0.133 (-0.071 to 0.337) |
|  | Female | **0.365 (0.100 to 0.630)** | 0.185 (-0.054 to 0.424) | **0.316 (0.120 to 0.512)** | **0.283 (0.101 to 0.465)** | **0.298 (0.133 to 0.464)** | **0.246 (0.063 to 0.428)** | **0.244 (0.077 to 0.412)** |
| Annual income | Male | 0.000 (-0.000 to 0.001) | 0.000 (-0.001 to 0.001) | 0.000 (-0.001 to 0.001) | -0.000 (-0.001 to 0.001) | -0.000 (-0.001 to 0.001) | -0.000 (-0.001 to 0.000) | 0.000 (-0.000 to 0.001) |
|  | Female | 0.001 (-0.001 to 0.002) | 0.000 (-0.001 to 0.001) | 0.001 (-0.001 to 0.002) | 0.001 (-0.000 to 0.003) | 0.000 (-0.001 to 0.001) | 0.000 (-0.001 to 0.002) | -0.000 (-0.002 to 0.001) |

**Panel C. DFLE-ADL**

| **Variable** | **Sex** | **2001** | **2004** | **2007** | **2010** | **2013** | **2016** | **2019** |
| --- | --- | --- | --- | --- | --- | --- | --- | --- |
| ***Disease burden indicators*** |  |  |  |  |  |  |  |  |
| Cardiovascular diseases | Male | NA | NA | NA | -0.000 (-0.001 to 0.001) | -0.001 (-0.002 to 0.000) | -0.000 (-0.001 to 0.001) | -0.000 (-0.001 to 0.001) |
|  | Female | NA | NA | NA | -0.001 (-0.002 to 0.000) | **-0.001 (-0.003 to -0.000)** | **-0.002 (-0.003 to -0.000)** | -0.001 (-0.002 to 0.000) |
| Neoplasms | Male | NA | NA | NA | -0.000 (-0.001 to 0.001) | 0.000 (-0.001 to 0.001) | 0.000 (-0.001 to 0.001) | -0.000 (-0.001 to 0.001) |
|  | Female | NA | NA | NA | -0.001 (-0.002 to 0.001) | -0.001 (-0.003 to 0.000) | -0.001 (-0.002 to 0.001) | -0.001 (-0.001 to 0.000) |
| Musculoskeletal disorders | Male | NA | NA | NA | 0.004 (-0.005 to 0.014) | 0.003 (-0.006 to 0.012) | 0.001 (-0.009 to 0.011) | 0.003 (-0.009 to 0.015) |
|  | Female | NA | NA | NA | 0.005 (-0.001 to 0.011) | 0.005 (-0.000 to 0.011) | 0.005 (-0.001 to 0.011) | 0.006 (-0.001 to 0.012) |
| Neurological disorders | Male | NA | NA | NA | -0.010 (-0.025 to 0.005) | -0.012 (-0.028 to 0.003) | -0.006 (-0.020 to 0.008) | -0.009 (-0.022 to 0.004) |
|  | Female | NA | NA | NA | -0.006 (-0.014 to 0.002) | -0.008 (-0.016 to 0.001) | -0.007 (-0.015 to 0.001) | -0.007 (-0.015 to 0.002) |
| Mental disorders | Male | NA | NA | NA | **-0.010 (-0.020 to -0.000)** | **-0.010 (-0.020 to -0.000)** | **-0.011 (-0.021 to -0.001)** | -0.009 (-0.019 to 0.000) |
|  | Female | NA | NA | NA | 0.001 (-0.004 to 0.007) | 0.001 (-0.004 to 0.007) | 0.001 (-0.004 to 0.006) | 0.002 (-0.003 to 0.006) |
| ***Risk factor exposures*** |  |  |  |  |  |  |  |  |
| High blood pressure | Male | NA | NA | NA | 0.005 (-0.073 to 0.082) | -0.001 (-0.094 to 0.091) | -0.021 (-0.132 to 0.091) | -0.034 (-0.147 to 0.080) |
|  | Female | NA | NA | NA | **0.127 (0.019 to 0.236)** | **0.109 (0.003 to 0.214)** | **0.234 (0.097 to 0.371)** | **0.254 (0.129 to 0.379)** |
| High fasting plasma glucose | Male | NA | NA | NA | 0.040 (-0.760 to 0.840) | 0.048 (-0.758 to 0.854) | 0.035 (-0.783 to 0.853) | 0.038 (-0.786 to 0.862) |
|  | Female | NA | NA | NA | -0.176 (-0.581 to 0.229) | -0.169 (-0.571 to 0.234) | -0.123 (-0.533 to 0.287) | -0.137 (-0.544 to 0.270) |
| High BMI | Male | NA | NA | NA | 0.746 (-1.912 to 3.403) | 0.673 (-1.952 to 3.299) | 0.791 (-1.858 to 3.440) | 0.717 (-1.967 to 3.401) |
|  | Female | NA | NA | NA | -0.453 (-2.234 to 1.328) | -0.554 (-2.341 to 1.234) | -0.550 (-2.332 to 1.233) | -0.513 (-2.311 to 1.285) |
| Tobacco | Male | NA | NA | NA | -0.052 (-0.380 to 0.277) | -0.004 (-0.340 to 0.332) | -0.020 (-0.359 to 0.319) | -0.088 (-0.433 to 0.256) |
|  | Female | NA | NA | NA | -0.094 (-0.310 to 0.122) | -0.065 (-0.299 to 0.169) | -0.071 (-0.322 to 0.180) | -0.127 (-0.390 to 0.137) |
| Dietary risk | Male | NA | NA | NA | -0.012 (-0.670 to 0.646) | -0.051 (-0.643 to 0.541) | -0.001 (-0.627 to 0.625) | 0.037 (-0.636 to 0.711) |
|  | Female | NA | NA | NA | -0.194 (-0.763 to 0.375) | -0.184 (-0.755 to 0.387) | -0.400 (-0.966 to 0.166) | -0.360 (-0.923 to 0.202) |
| ***Socioeconomic and healthcare indicators*** |  |  |  |  |  |  |  |  |
| Per capita medical exp | Male | NA | NA | NA | -0.008 (-0.018 to 0.003) | -0.006 (-0.016 to 0.005) | -0.008 (-0.019 to 0.003) | -0.006 (-0.017 to 0.005) |
|  | Female | NA | NA | NA | 0.002 (-0.009 to 0.012) | 0.000 (-0.010 to 0.011) | 0.003 (-0.007 to 0.012) | 0.002 (-0.007 to 0.012) |
| Nursing homes per 100k | Male | NA | NA | NA | -0.004 (-0.012 to 0.004) | -0.002 (-0.008 to 0.003) | -0.001 (-0.006 to 0.003) | 0.003 (-0.001 to 0.007) |
|  | Female | NA | NA | NA | 0.002 (-0.008 to 0.012) | -0.000 (-0.007 to 0.006) | 0.002 (-0.004 to 0.008) | 0.002 (-0.001 to 0.006) |
| Labor force ratio (%) | Male | NA | NA | NA | 0.013 (-0.044 to 0.071) | 0.045 (-0.024 to 0.114) | 0.026 (-0.031 to 0.083) | 0.025 (-0.024 to 0.073) |
|  | Female | NA | NA | NA | -0.059 (-0.132 to 0.015) | -0.048 (-0.111 to 0.016) | -0.037 (-0.104 to 0.030) | -0.049 (-0.113 to 0.015) |
| University grad ratio | Male | NA | NA | NA | 0.016 (-0.070 to 0.103) | 0.024 (-0.058 to 0.105) | -0.000 (-0.086 to 0.085) | 0.008 (-0.073 to 0.090) |
|  | Female | NA | NA | NA | 0.074 (-0.099 to 0.247) | 0.083 (-0.077 to 0.243) | 0.076 (-0.054 to 0.206) | 0.092 (-0.023 to 0.206) |
| Pop aged 75+ (%) | Male | NA | NA | NA | **0.175 (0.049 to 0.301)** | 0.072 (-0.051 to 0.194) | **0.182 (0.046 to 0.318)** | **0.183 (0.052 to 0.314)** |
|  | Female | NA | NA | NA | **0.090 (0.009 to 0.171)** | **0.093 (0.004 to 0.182)** | 0.078 (-0.005 to 0.160) | **0.100 (0.023 to 0.177)** |
| Annual income | Male | NA | NA | NA | -0.000 (-0.000 to 0.000) | -0.000 (-0.001 to 0.000) | 0.000 (-0.000 to 0.000) | 0.000 (-0.000 to 0.000) |
|  | Female | NA | NA | NA | 0.000 (-0.000 to 0.001) | -0.000 (-0.001 to 0.001) | 0.000 (-0.000 to 0.001) | -0.000 (-0.001 to 0.000) |

CI = confidence interval; DFLE-AL = disability-free life expectancy based on activity limitation; LE-SH = life expectancy in self-perceived health; DFLE-ADL = disability-free life expectancy based on activities of daily living; GBD = Global Burden of Disease; HALE = healthy life expectancy; MHLW = Ministry of Health, Labour and Welfare.

*Note: DFLE-ADL data are available from 2010 onwards. Bold values indicate statistical significance (95% CI does not include zero).*
